# Research on the Mechanism of Asperosaponin VI for Treating Recurrent Spontaneous Abortion by Bioinformatics Analysis and Experimental Validation

**DOI:** 10.1155/2022/8099853

**Published:** 2022-06-23

**Authors:** Bo Xia, Peng Zhang, Yuling Lai, Shichao Cui, Zhenyue Chen, Qingying Yu, Haiwang Wu, Lihua Zeng, Baozhen Xie, Jingwei Li, Huimin Zhang, Songping Luo, Jie Gao

**Affiliations:** ^1^Guangzhou University of Chinese Medicine, Guangzhou 510405, China; ^2^The First Aﬃliated Hospital of Guangzhou University of Chinese Medicine, Guangzhou 510405, China; ^3^Lingnan Medical Research Center of Guangzhou University of Chinese Medicine, Guangzhou 510405, China

## Abstract

Asperosaponin VI (AS6), as the quality marker of *Dipsaci Radix*, is verified to exert therapeutic effect on alleviating recurrent spontaneous abortion (RSA). However, due to the lack of relevant research, its molecular mechanism is still unclear. We retrieved targets for AS6 and RSA, and then used their overlapped targets for PPI analysis. In addition, we used GO and KEGG enrichment analyses, and molecular docking to investigate the anti-RSA mechanisms of AS6. Furthermore, we conducted *in vitro* experiments to validate the predictions of network pharmacology. Results showed that a total of 103 AS6-associated targets and 2084 RSA-associated targets, with 49 targets overlapped. GO enrichment analysis showed 845 significant biological processes like decidualization, while KEGG pathway enrichment analysis revealed 76 significant entries including 18 signaling pathways, which were closely linked to PI3K-Akt, HIF-1, TNF, IL-17, and VEGF signaling pathways, etc. Molecular docking findings verified that AS6 had tight link with the key targets including JUN, CASP3, STAT3, SRC, and PTGS2. Notably, *in vitro* experiments revealed that AS6 treatment could exert lower expressions of JUN, pro-CASP3, CASP3, STAT3, SRC, and PTGS2 in decidual cells compared with progesterone despite the expressions of STAT3, SRC, and PTGS2 with no significant difference, and mifepristone could interfere with the effects. In general, numerous targets and multiple pathways involve during the process of AS6 treatment against RSA. Moreover, our *in vitro* research first reported that AS6 may regulate the expressions of key targets (JUN, CASP3, STAT3, SRC, and PTGS2) in decidual cells to promote decidualization, thus treating RSA.

## 1. Introduction

Recurrent spontaneous abortion (RSA) is a common reproductive endocrine disorder, which refers to consecutive spontaneous abortions happening twice or more before 20 weeks of gestation [1, 2]. According to statistics, over 5% of women at reproductive age suffer from RSA [3]. Multiple pathological factors lead to RSA, including genetic factors, anatomical abnormalities, endocrine disorders, infectious diseases, and thyroid dysfunction [4, 5]. Embryonic chromosomal abnormality is the main cause of spontaneous abortion in early pregnancy, which results in over 50% of first trimester miscarriages [6–8]. However, there exist approximately 50% of RSA cases with unexplained recurrent spontaneous abortion [9]. RSA seriously poses threat to the life and health of pregnant women, increasing the burden on families and society. In recent years, the therapeutic effects of traditional Chinese medicine (TCM) on RSA have gradually been proven, attracting the attention of more and more scholars [10–12].

Shoutai pill has been often used for treating unexplained recurrent spontaneous abortion in China [10]. It has been revealed that Shoutai pill can maintain the balance of Th1/Th2 cytokines, which improves endometrial receptivity and embryo implantation thus exerting therapeutic effects on RSA [13]. To date, the hot spots of current researches focus on the active ingredients of Shoutai pill. Asperosaponin VI (AS6, Pubchem CID: 118705380) is the quality marker of *Dipsaci Radix*, which is an important drug in Shoutai pill. Pharmacological studies in recent decades have shown that *Dipsaci Radix* has a variety of biological activities, including antiuterine contraction, antiinflammatory, antiaging, antiarthritic, antiosteoporosis, fracture healing, neuroprotection, and it has been verified to benefit Chinese women from miscarriages, serving as the preferred herb in clinical treatment [14]. According to Chinese Pharmacopoeia 2020 edition, *Dipsaci Radix* exerts effects on tocolysis and uterine bleeding during pregnancy, which has been an accumulated experience for thousands of years [15]. To date, the active components isolated from *Dipsaci Radix* mainly include saponins, triterpenes, volatile oils, and alkaloids, which may have curative effects on female reproductive disorders through significantly suppressing the spontaneous contractions of the gestational uterus induced by oxidative toxins [16]. Existing evidence suggests that *Dipsaci Radix* has an important application in anti-RSA treatment [15]. Moreover, we have investigated the action mechanism of *Dipsaci Radix* extracts and AS6 in our previous research and have observed that they may exert therapeutic effects on RSA by activating decidual cells' progesterone receptor expression through Notch signaling pathway [17]. The angiogenesis disorders of the endometrium and infection play important part in RSA, and existing evidence has shown that AS6 efficiently accelerates the angiogenesis of regenerated tissue and facilitates wound healing *in vivo*, and improves vascularization of human umbilical vein endothelial cells (HUVECs) *in vitro* by the upregulation of HIF-1*α*/VEGF pathway [18]. Moreover, it has been revealed that AS6 also inhibits the morphological expansion of microglia cells, decreases the expression, and releases of proinflammatory cytokines, such as IL-1B, iNOS, IL-6, and TNF-a in a dose-dependent manner [19]. However, whether AS6 can treat RSA through these pathways needs further research.

In the present study, we carried out bioinformatics analysis integrated with experimental validation so as to perform systematic analysis on various targets and pathways of AS6 for treating RSA.

## 2. Methods

**Figure 1:**
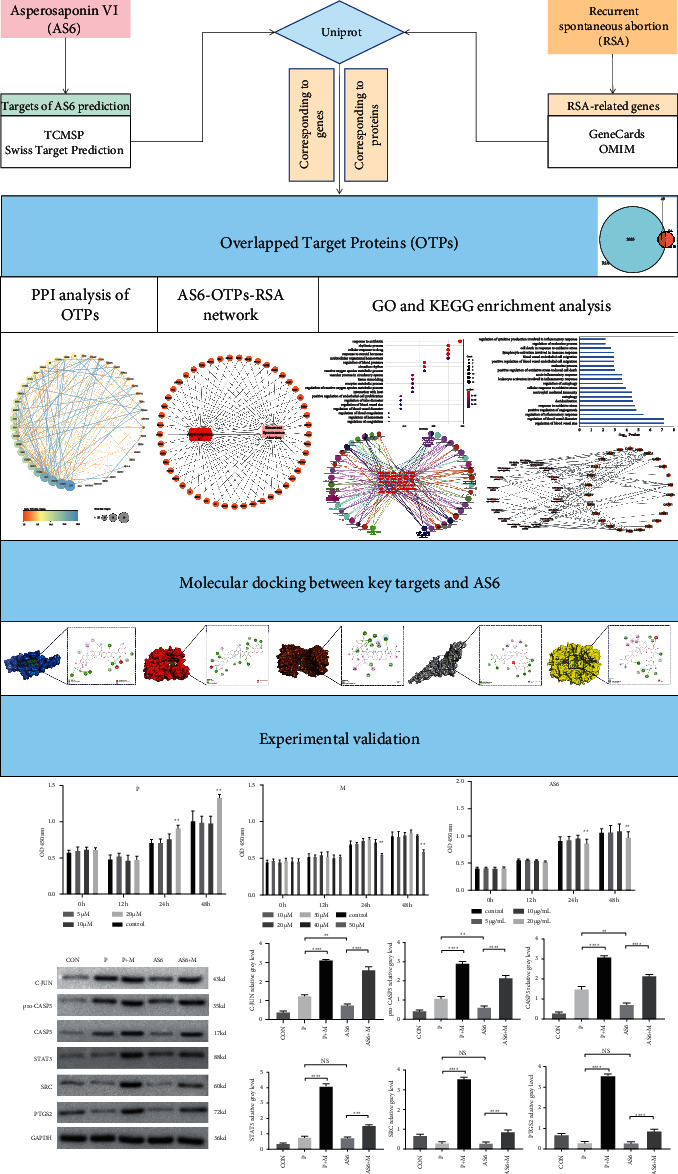
The flow chart of this study.

We have referred to the methods of Ren et al. [20].  Figure 1 described the study flowchart.

### 2.1. Data Retrieval of Network Pharmacology

#### 2.1.1. Retrieval of AS6-Associated Structure and Targets

First, we retrieved the information of AS6-associated structure and targets by searching TCMSP platform (https://tcmsp-e.com/) [21]. Second, Pubchem website (https://pubchem.ncbi.nlm.nih.gov/) was used to obtain the AS6 structure stored as “SDF” file that we subsequently uploaded into SwissTargetPrediction platform (https://new.swisstargetprediction.ch/) [22] to get the targets associated with AS6. Third, we used the UniProt platform (https://www.uniprot.org/uniprot/) to standardize the AS6-associated target information restricted to humans.

#### 2.1.2. Retrieval of *RSA-Associated* Genes and Their Corresponding Proteins

Taking “Recurrent Spontaneous Abortion” as the key word, we searched GeneCards (https://www.genecards.org/) [23] and Online Mendelian Inheritance in Man (OMIM, https://omim.org/) [24], respectively. Then we carried out data standardization through Uniprot database to obtain corresponding proteins of *RSA-associated* genes.

#### 2.1.3. Overlapped Target Proteins (OTPs)

We utilized *R* (v3.6.1) software to take the intersection of AS6- and RSA-associated targets to obtain OTPs.

### 2.2. Data Analysis of Network Pharmacology

#### 2.2.1. OTPs-Associated Protein Interaction Analysis

We obtained OTPs-associated Protein–Protein Interaction (PPI) data via retrieving the STRING platform (https://string-db.org/) [25]. Next, we plotted the PPI network with the PPI information of OTPs imported into Cytoscape software (v3.7.2; https://www.cytoscape.org/) [26] and carried out network topology analysis for the calculation of the target degrees. We screened the core targets with degrees above average. Afterwards, we constructed an AS6-OTPs-RSA network via Cytoscape.

#### 2.2.2. GO Enrichment Analysis and KEGG Pathway Analysis

We performed Gene Ontology (GO) enrichment analysis concerning biological process (BP) via clusterProfiler package (R3.6.1) and selected the enrichment results with *p* < 0.05. Then the top 20 items and 20 representative items closely related to the pathological process of RSA were presented. Additionally, we input OTPs into Cytoscape for GO.BP enrichment analysis with *p*-value set to 0.001, and performed network visualization to establish linkages between biological processes and targets. Next, we carried out Kyoto Encyclopedia of Genes and Genomes (KEGG) analysis of OTPs using clusterProfiler package (R3.6.1), extracted the significant enrichment results (*p* < 0.05), and plotted pathway-target network using Cytoscape.

### 2.3. Molecular Docking between Key Targets and AS6

We selected the top five proteins in terms of degree for molecular docking, which were recognized as the key targets in the treatment of AS6 for RSA. We adopted AutoDock Vina software (v1.1.2) [27] to perform molecular docking simulations to investigate interaction activities between AS6 and key targets. The 3D structure of AS6 was obtained by retrieving the Pubchem platform (https://pubchem.ncbi.nlm.nih.gov/). AutoDock Tools (v1.5.6) was utilized to distribute charge and combine nonpolar hydrogen for AS6 and convert the results into a PDBQT file. We searched RCSB PDB website (https://www.rcsb.org/) for the crystal structures of key targets. Then the target protein was separated from its ligand, distributed charge, and added polar hydrogen via AutoDock Tools, which would be subsequently stored as a PDBQT file. We used AutoDock Tools to determine the size and center of the docking box. Afterwards, we performed molecular docking simulations among AS6 and the target proteins with every affinity calculated. Then we analyzed and plotted the docking results of AS6 via PyMol and Discovery Studio.

### 2.4. Validation of AS6 by *in Vitro* Assays

#### 2.4.1. Cells, Reagents, and Antibodies

We obtained primary decidua cells from decidua tissues. The source of Asperosaponin VI was purchased from Guangdong Food and Drug Administration, China. We purchased progesterone and mifepristone from Sigma Aldrich. We bought 0.25% trypsin, DMEM/F12, FBS, Charcoal dextran-treated FBS, and Lipofectamine™ 2000 from Gibco. The antibodies used in this research, such as C-JUN (AF1612), CASP3 (AC030), STAT3 (AF1492), SRC (AF1831), PTGS2 (AF1924), and GAPDH (AF1186) antibodies were purchased from Beyotime (Shanghai, China). We diluted the primary antibody at a ratio of 1 : 1000 with QuickBlock™ Primary Antibody Dilution Buffer for Western Blotting (Beyotime) and the secondary antibody at a ratio of 1 : 10000 with QuickBlock™ Secondary Antibody Dilution Buffer for Western Blotting (Beyotime).

#### 2.4.2. Isolation and Culture of Primary Decidual Cells

We obtained decidua samples at 6–9 week of gestation from singleton pregnant women who requested normal pregnancy termination or who underwent excretion of retained pregnancy products after a failed spontaneous pregnancy. All patients signed written informed consent in accordance with Declaration of Helsinki, and permission was obtained from Ethics Committee of the 1st Affiliated Hospital of Guangzhou University of TCM. The ethics code is No.K [2019] 098.

Fresh decidua tissues were taken aseptically, washed in PBS to remove blood, cut into pieces, digested with trypsin-EDTA (0.25%) for 5–10 min, and digestion was stopped by adding DMEM/F12 medium containing 10% FBS. The cell clusters in the final digestion were extracted with a 23-gauge needle, filtered through a 200-mesh sieve, centrifuged at 2000 rpm for 5 min, resuspended in DMEM/F12 medium containing 10% FBS, and incubated in flask at 5 × 10^5^ cells/ml. After 30 min, we removed nonadherent cells and replaced the medium after 48 h.

#### 2.4.3. Cell Counting Kit 8 Assay

We treated the decidual cells with a concentration gradient of progesterone (P), mifepristone (M), and AS6 for 12, 24, and 48 h. According to the manufacturer's protocol, we performed a cell counting kit 8 (CCK-8) assay to detect cell proliferation abilities using an optical density (OD) setting of 450 nm in the microplate reader (Varioskan Flash; Thermo Fisher Scientific, Waltham, MA, USA).

#### 2.4.4. Western Blotting

We cultured decidual cells in 6-well plates and treated them with the specific concentration of progesterone, Asperosaponin VI with or without mifepristone according to CCK-8 screening results. After the treatment, the protein was extracted by adding 200 *μ*L RIPA lysis buffer prepared with phosphatase inhibitor and protease inhibitor per dish (Beyotime). The protein bands were transferred to polyvinylidene fluoride membranes (Shanghai, microtiter wells) by electrophoresis and wet transfer steps, closed with QuickBlock™ Western closing solution (Beyotime) at room temperature for 30 min, added primary antibody and incubated overnight at 4°C in a shaker, then added the corresponding secondary antibody and incubated in shaker at 24°C for 1.5 h. The antibody reactivity level was subsequently detected by gel imaging system (Bio-Rad). Finally, the grayscale values were quantitated using ImageJ software.

#### 2.4.5. Statistical Analysis

All results were expressed as mean ± standard deviation. Student's *t*-tests were used to compare two separate samples. One-way ANOVA was used for comparison of univariate samples between multiple groups. *p*-value <0.05 indicates statistical significance.

## 3. Results

### 3.1. AS6-Associated Structure and Target Proteins

We obtained a total of 103 AS6-associated targets. After data standardization by the UniProt database, we obtained AS6-related target proteins called as Gene symbols. AS6-associated structure and target results were shown in supplementary Tables S1–S2.

### 3.2. RSA-Associated Target Information and Overlapped Target Proteins (OTPs)

**Figure 2:**
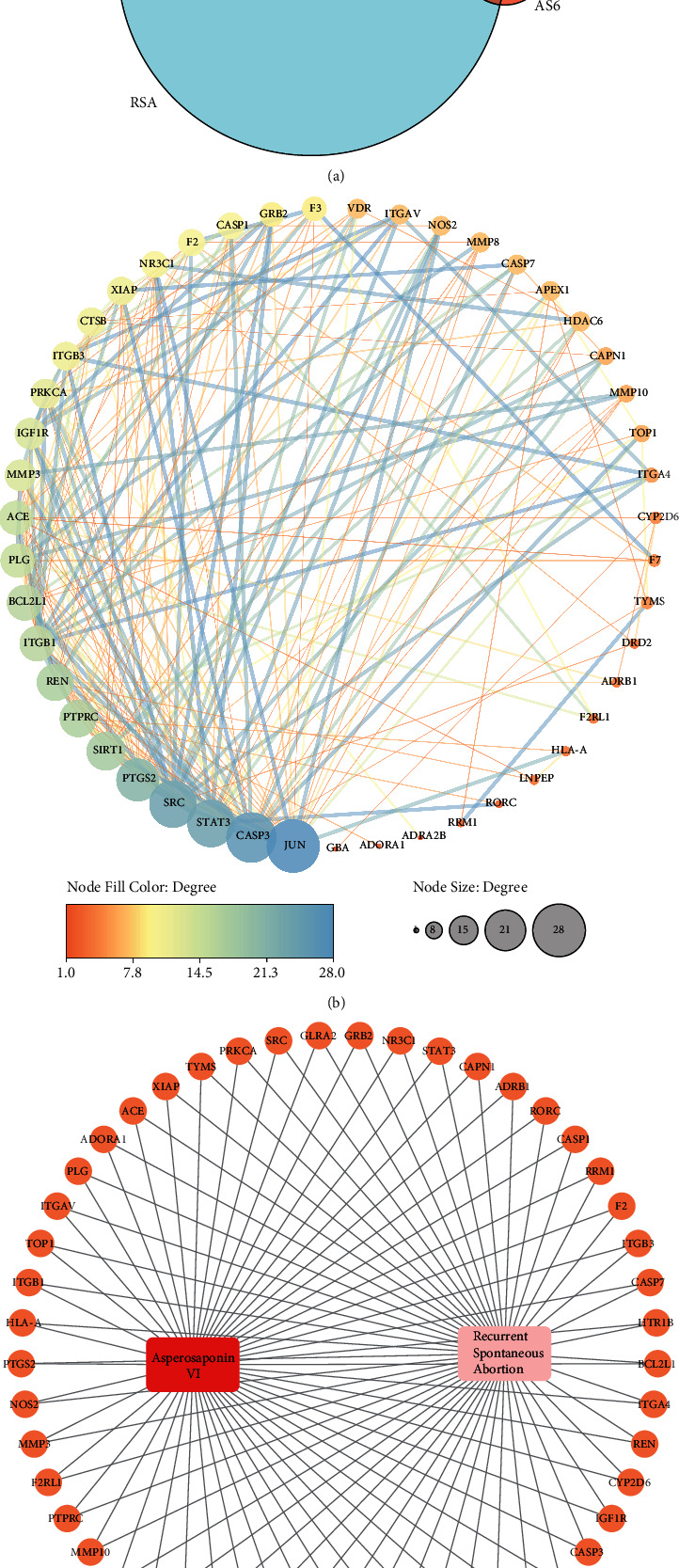
Venn diagram of OTPs (a), PPI network of OTPs (b), and AS6-OTPs-RSA network (c).

A total of 2084 RSA-associated target proteins were retrieved. We took the overlap of AS6- and RSA-associated targets as OTPs, which included 49 overlapped targets, as shown in Table 1 and Figure 2(a).

### 3.3. Construction of PPI Network and Screening Core Target Proteins

OTPs were imported into the STRING platform with the targets having no link to others hidden. We imported the PPI data into Cytoscape to draw PPI network in Figure 2(b). There were 21 target proteins predicted to be the core target proteins (Table 2), whose degrees were above average degree (9.83).

### 3.4. AS6-OTPs-RSA Network Plotting

Figure 2(c) shows AS6-OTPs-RSA network with 51 nodes and 98 edges included. In Figure 2(c), the orange circular nodes stand for the overlapped target proteins (OTPs). The red rectangle node stands for “Asperosaponin VI.” The pink rectangle node stands for “Recurrent Spontaneous Abortion.” The edges stand for the interactive relationships between Asperosaponin VI, recurrent spontaneous abortion, and the overlapped targets.

### 3.5. GO Enrichment Analysis

**Figure 3:**
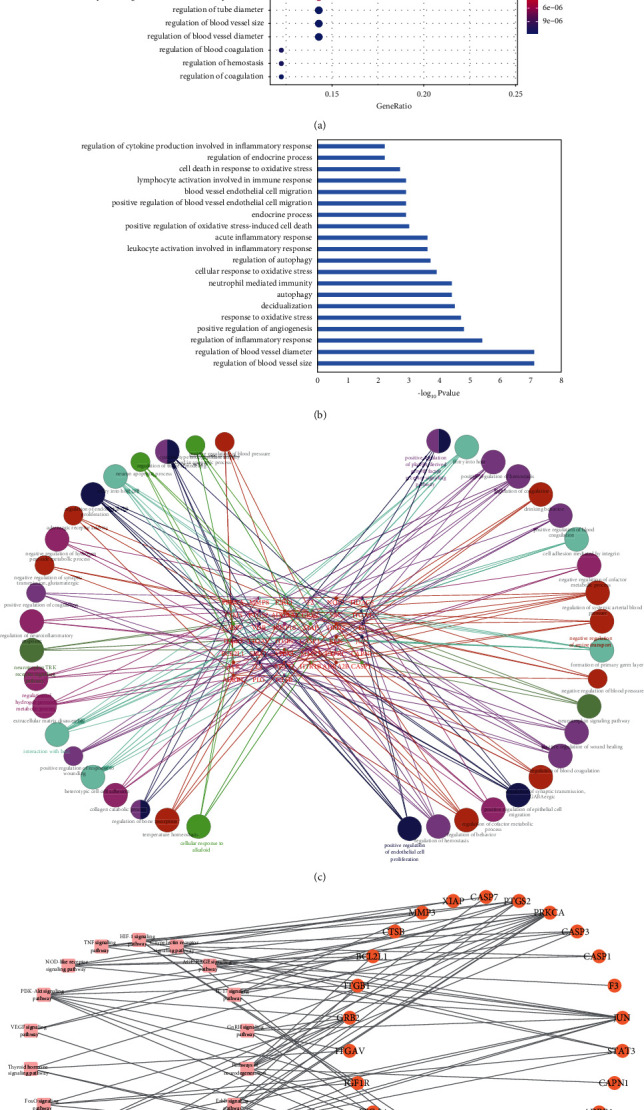
GO.BP enrichment analysis (a–c), and pathway-target network (d). (a,b) The top and screened 20 items of biological processes in terms of p-value. (c) Different colors represent different biological process groups and node size stands for term p-value, while the edges represent the connections between biological processes and targets. (d) A pink square node represents a signaling pathway, an orange circular node represents a gene, and an edge represents a relationship between a pathway and a gene.

We got 845 items of biological process (BP). The top 20 items were shown in Figure 3(a). Noteworthily, we have filtrated 20 items mainly linked to autophagy, blood vessel endothelial cell migration, angiogenesis, inflammatory response, oxidative stress, decidualization, endocrine process, and immune response, which were demonstrated in Figure 3(b). Additionally, we input 49 OTPs into Cytoscape for GO.BP enrichment analysis with *p*-value set to 0.001. Figure 3(c) illustrated the enrichment results mainly involved in four aspects as follows: (i) inflammation-related activities, such as regulation of neuroinflammatory response and extracellular matrix disassembly which is closely associated with oxidative stress; (ii) cell cycle, such as positive regulation of endothelial cell proliferation and migration; (iii) tissue repair, such as positive regulation of response to wounding and wound healing and regulation of tissue remodeling; and (iv) endocrine metabolism process, such as regulation of cofactor metabolic process, adrenergic receptor activity, and negative regulation of synaptic transmission.

### 3.6. KEGG Pathway Analysis

We finally got totally 76 items including 18 key signaling pathways listed in Table 3. These signaling pathways such as PI3K-Akt, HIF-1, TNF, IL-17, and VEGF may exert regulatory functions on the process of AS6 against RSA. We conducted network visualization via Cytoscape as plotted in Figure 3(d), which established the relationship between signaling pathways and targets. Specifically, several OTPs were involved in PI3K-Akt signaling pathway (e.g., BCL2L1, ITGB1, GRB2, PRKCA, ITGAV, IGF1R, ITGA4, ITGB3), HIF-1 signaling pathway (e.g., STAT3, NOS2, PRKCA, and IGF1R), TNF signaling pathway (e.g., PTGS2, JUN, CASP3, CASP7, and MMP3), IL-17 signaling pathway (e.g., PTGS2, JUN, CASP3, and MMP3), and VEGF signaling pathway (e.g., PTGS2, SRC, PRKCA).

### 3.7. Molecular Docking Analysis

Among 21 core targets, the top five target proteins in terms of degree were chosen for molecular docking, including JUN, CASP3, STAT3, SRC, and PTGS2, respectively, which were considered as the key targets in the process of AS6 treating RSA. To verify how AS6 binds to the key targets, we adopted molecular docking using Autodock Vina to predict their docking interactions.  Table 4 showed the docking results including affinity and interaction information.

**Figure 4:**
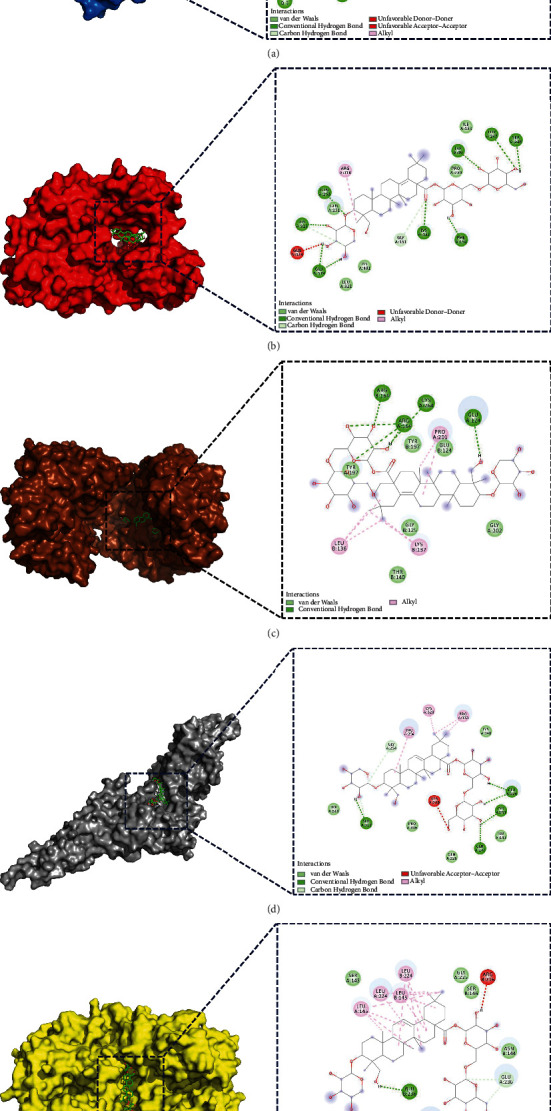
Simulated molecular docking of Asperosaponin VI on JUN (a), SRC (b), CASP3 (c), STAT3 (d), and PTGS2 (e).

Based on Figure 4(a), AS6 combined with JUN by forming six hydrogen bonds with the residues including Gln-30, Arg-5, Arg-21, Asp-26, and Lys-22 (binding affinity: −7.2 kcal/mol). Besides, there were three van der Waals interactions between AS6 and Tyr-18, Lys-9, and Leu-13.

Based on Figure 4(b), the docking affinity of AS6 on SRC was −9.3 kcal/mol. The residues containing Glu-339, His-319, Gln-253, Lys-152, Phe-150, Tyr-90, Thr-247, and Ser-248 linked to AS6 by forming nine hydrogen bonds, which provided a powerful electrostatic force for the combination of AS6 and SRC. Moreover, there were five van der Waals interactions between AS6 and Gln-251, Leu-322, Lys-401, Pro-250, and Ile-153.

Based on Figure 4(c), the docking affinity of AS6 on CASP3 was −9.7 kcal/mol. There existed six hydrogen bonds provided by the Arg-164, Cys-264, and Glu-124 residues in the link to AS6. Moreover, AS6 binded with the Gly-125, Thr-140, Gly-202, Tyr-197, and Glu-124 residues by six van der Waals.

Based on Figure 4(d), the docking affinity of AS6 on STAT3 was −7.6 kcal/mol. There were five hydrogen bonds provided by the Glu-324, Ser-513, Gln-247, and Cys-251 residues in the interaction with AS6. What is more, there were five van der Waals interactions between AS6 and Trp-510, Pro-336, Lys-348, Gln-326, and Trp-243.

Based on Figure 4(e), the docking affinity of AS6 on PTGS2 was −10.5 kcal/mol. The Glu-236, Ser-143, and Glu-140 residues formed three hydrogen bonds in the interaction with AS6. Additionally, there were five van der Waals interactions between AS6 and Ser-143, Arg-333, Asn-144, Ser-146, and Gly-225.

### 3.8. CCK-8 Assay

**Figure 5:**
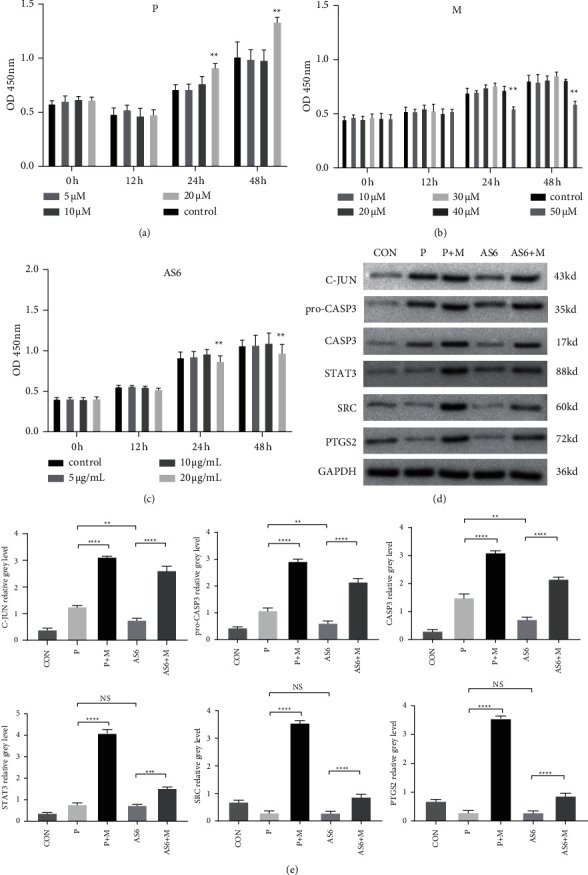
Asperosaponin VI influenced key targets' expression in decidual cells. Decidual cells were treated with progesterone (P), or Asperosaponin VI (AS6) respectively, with or without mifepristone (M) for 24 h in Western blotting. (a, b, c) CCK-8 assays of (P) M and AS6 (d) The protein expressions of key targets including JUN, CASP3, STAT3, SRC and PTGS2 were detected by Western blotting. (e) Representation of the relative grey level in (D) Data are displayed as mean ± standard deviation. ^*∗∗*^*p* < 0.01; ^*∗∗∗*^*p* < 0.001; ^*∗∗∗∗*^*p* < 0.0001.

We performed CCK-8 assays before the *in vitro* research. The concentrations of progesterone used in the study were 0 (control group), 5, 10, and 20 *μ*mol/L. The results revealed that the progesterone concentration at 20 *μ*mol/L exerted proliferative effect on the proliferation of decidual cells, which was selected for subsequent experiments (Figure 5(a)). The concentrations of mifepristone used in the study were 0 (control group), 10, 20, 30, 40, and 50 *μ*mol/L. The results revealed that the mifepristone concentration at 50 *μ*mol/L exerted suppressive effect on the proliferation of decidual cells, which was selected for subsequent experiments (Figure 5(b)). The concentrations of AS6 used in the study were 0 (control group), 5, 10, and 20 *μ*g/mL. The results revealed that there was no cytotoxicity to decidual cells when the AS6 concentration was no higher than 10 *μ*g/mL with the proliferation of decidual cells neither promoted nor inhibited, which was selected for subsequent experiments (Figure 5(c)).

### 3.9. Western Blotting Analysis

To investigate the function of Asperosaponin VI in decidual cells, we tested the expression levels of specific proteins including JUN, CASP3, pro-CASP3, STAT3, SRC, and PTGS2 to examine the influence of Asperosaponin VI treatment via Western blotting. The treatment concentrations of progesterone, mifepristone, and Asperosaponin VI were 20 *μ*mol/L, 50 *μ*mol/L, and 10 *μ*g/mL respectively, according to CCK-8 assay. As shown in Figures 5(d)–5(e), Asperosaponin VI treatment could exert lower expressions of JUN, pro-CASP3, CASP3, STAT3, SRC, and PTGS2 in decidual cells compared with progesterone, while the expressions of STAT3, SRC, and PTGS2 showed no significant difference between Asperosaponin VI-treated and progesterone-treated groups, and mifepristone could interfere the effects.

## 4. Discussion

Chinese traditional medicine *Dipsaci Radix*, a drug in Shoutai pills, has been widely applied in treating gynecological diseases like RSA clinically for many years. Our present study explored the mechanisms of Asperosaponin VI in treating RSA, which is an important component of *Dipsaci Radix*.

Progesterone (P) exerts essential effects on the maintenance of pregnancy, the declining level of which in blood in early pregnancy leads to necrosis of the decidua, thereby causing miscarriage [28]. Mifepristone (M) is the first-known progesterone antagonist, which eventually results in conception abortion when used postimplantation [29]. In this study, we reported the strong progesterone-like effects of Asperosaponin VI and its actions in the treatment of RSA.

According to PPI network topology analysis of OTPs, we noticed that these targets were characteristics of decidualization, autophagy, angiogenesis, oxidative stress, inflammation, and endocrine-related proteins. We identified five key targets including JUN, CASP3, STAT3, SRC, and PTGS2, which are in close conjunction with AS6 according to molecular docking findings, indicating that they may be the key targets of AS6 in treating RSA.

JUN (Transcription factor AP-1 subunit Jun), which is the mediator of trophoblast invasion, plays a critical role in decidualization [30, 31]. It has been revealed that downregulation of JUN production could alter epithelial mesenchymal transition (EMT)-related molecule expression, which would impede trophoblast migration and invasion [32]. Existing research has confirmed that activation of JUN expression involves the chemokine recruitment of human first trimester decidual cells (FTDCs), triggering response to proinflammatory stimuli, which serves as an essential factor for RSA [33]. Further study has clarified that the accumulation of CX3CL1 chemokine results from the induction of IL-1*β*, TNF-*α*, and IFN-*γ* in FTDCs, which can be mediated by the activation of JUN-related signaling [34]. Notably, our experiments displayed lower expression level of JUN in decidual cells in AS6-treated group compared with progesterone-treated group, suggesting that AS6 could suppress the expression of JUN in decidual cells to promote decidualization so as to anti RSA.

Caspase-3 (CASP3) is an apoptosis-related gene, whose expression has close correlation with placental separation [35]. Some studies have identified myometrial CASP3 as a potential regulator of uterine quiescence, and uterine endoplasmic reticulum stress-unfolded protein response regulation of gestational length is CASP3-dependent [36]. Decidual cell apoptosis could be mediated by TNF-related apoptosis-induced ligand (TRAIL) via CASP3-dependent pathway, whose expression is upregulated in decidua from women suffering from RSA [37]. Moreover, the inhibition of CASP3 activity could prevent the apoptosis of uterine stromal cells, which could proliferate and then differentiate into decidual cells during the process of decidualization [38]. CASP3 exerts an essential role during the process of decidualization, while the increased expression of CASP3 in endometrium decidua indicates poor endometrial receptivity, which could lead to RSA [39]. Notably, our experiments revealed that AS6 treatment could exert lower expressions of pro-CASP3 and CASP3 in decidual cells compared with progesterone, suggesting that AS6 could downregulate CASP3 expression in decidual cells to promote decidualization so as to anti RSA.

Signal transducer and activator of transcription 3 (STAT3) phosphorylation has a close relationship with embryo implantation and decidualization [40]. RSA results from impaired trophoblast function, and further study has shown that STAT3 expression could affect trophoblast cell proliferation and migration [41]. Existing studies have confirmed that the reduction of plasmacytoid dendritic cells in RSA could be mediated by the regulation of STAT3 expression [42]. STAT3 signaling has been verified to exert anti-inflammatory IL-10 expression in decidua cells to protect pregnancy [43]. SRC (Proto-oncogene tyrosine-protein kinase Src) is endometrial nuclear receptor cofactor, which plays an important part in regulating human endometrium remodeling [44]. It has been shown that SRC could regulate endometrial function and progesterone-related gene expression [45]. Transcriptomics has confirmed that SRC gets involved in the process of decidualization [46]. Further study has shown that the expression of SRC is necessary for invasion and migration of human decidual stromal cells, which exerts vital functions in embryo implantation and human pregnancy [47]. Prostaglandin G/H synthase 2 (PTGS2) is related to the regulation of inflammatory response, the regulation of which influences the decidualization response of endometrial stromal cells [48]. Numerous studies have shown that PTGS2 is identified as important regulators of early pregnancy events and plays a vital role in human decidualization and vascularization of the endometrial stroma [49, 50]. In our present study, we observed lower expressions of STAT3, SRC, and PTGS2 in decidual cells after AS6 treatment compared with progesterone, but the difference was not statistically significant, suggesting that AS6 may exert progesterone-like effect in the treatment of RSA.

Similar to PPI analysis, GO enrichment results show consistent results as demonstrated in Figure 3(b). Decidualization plays an indispensable role in normal pregnancy, while suppressed decidualization contributes to increased prevalence of RSA [51]. Numerous studies have confirmed that the expressions of key targets including JUN [52], CASP3 [38], STAT3 [53], SRC [46], and PTGS2 [54] play an essential role in decidualization. In the present study, we observed that AS6 displayed strong effects on the expressions of JUN, CASP3, STAT3, SRC, and PTGS2, even better than the positive control progesterone, indicating that AS6 may play a strong progesterone-like function to promote decidualization against RSA. It has been verified that autophagy makes key functions in RSA-related pathogenesis, which affects trophoblast invasion and adhesion [55]. Some evidences have illustrated that oxidative stress is one of the important factors that trigger RSA [56]. According to our present study, AS6 may be an antioxidant with a good prospect that helps reduce oxidative stress and improve RSA. Endometrial angiogenesis disorders and infection exert key functions in RSA, and it has been shown that AS6 can effectively accelerate the angiogenesis of regenerated tissues and promote wound healing, and promote the vascularization of HUVECs [18]. AS6 can also inhibit the morphological expansion of microglia, reduce the expression of pro-inflammatory cytokines such as IL-1B, iNOS, TNF-*α*, IL-6, IL-1B, and TNF-*α* in a dose-dependent manner [19].

KEGG enrichment results revealed that PI3K-Akt, HIF-1, TNF, IL-17, and VEGF signaling pathways may exert regulatory functions on AS6 against RSA. Some studies have verified that the inhibition of PI3K-Akt signaling pathway can reduce trophoblast cell proliferation and migration [57]. Moreover, studies have shown that activation of PI3K-Akt signaling pathway could promote endometrial decidualization [58]. However, whether AS6 could regulate PI3K-Akt signaling pathway to treat RSA is still unclear, which needs further identification in the future research. Our present study has shown that AS6 may treat RSA through the regulation of angiogenesis and tissue repair as described in Figure 3(b). And some studies have verified that AS6 can promote the angiogenesis of HUVECs *in vitro* by upregulating HIF-1*α*/VEGF pathway and can effectively promote the angiogenesis of regenerative tissues and promote wound healing *in vivo* [18]. So HIF-1 signaling pathway and VEGF signaling pathway have close connection with AS6 treatment in RSA. In addition, inflammatory response-related pathways including TNF and IL-17 signaling pathways play vital role in the pathological process of RSA. Existing study has shown that the balance between pro-inflammatory cytokines on TNF signaling pathway exerts important influence on the success or failure of the implanted embryos [59]. Abnormal expression of IL-17 in the feto-maternal interface may lead to RSA [60].

In summary, our results predict some potential therapeutic targets and pathways, providing reference for future studies on AS6 treatment against RSA. However, one limitation of this study is that further *in vivo* and *in vitro* experiments are needed to confirm our findings.

## 5. Conclusion

Collectively, our results revealed that AS6 may treat RSA possibly by regulating numerous signaling pathways and targets related with decidualization, autophagy, blood vessel endothelial cell migration, angiogenesis, inflammatory response, oxidative stress, and immune response, etc. Moreover, our *in vitro* study first reported that AS6 may regulate the expressions of key targets in decidual cells including JUN, CASP3, STAT3, SRC, and PTGS2 to promote decidualization, thus treating RSA.

## Figures and Tables

**Table 1:** Potential target genes of AS6 in the treatment of RSA.

Number	Gene	Number	Gene
1	BCL2L1	26	PTPRC
2	F2	27	F3
3	RORC	28	F7
4	STAT3	29	LNPEP
5	GLRA2	30	CTSB
6	TYMS	31	CASP3
7	ADORA1	32	REN
8	TOP1	33	CASP7
9	PTGS2	34	CASP1
10	F2RL1	35	CAPN1
11	GBA	36	GRB2
12	JUN	37	PRKCA
13	VDR	38	ACE
14	ADRA2B	39	ITGAV
15	DRD2	40	HLA-A
16	CYP2D6	41	MMP3
17	HTR1B	42	MMP10
18	RRM1	43	SIRT1
19	ADRB1	44	MMP8
20	NR3C1	45	APEX1
21	SRC	46	HDAC6
22	XIAP	47	IGF1R
23	PLG	48	ITGA4
24	ITGB1	49	ITGB3
25	NOS2		

**Table 2:** Core targets of AS6 in the treatment of RSA.

Number	Core Targets	Degree
1	JUN	28
2	CASP3	26
3	STAT3	24
4	SRC	24
5	PTGS2	21
6	SIRT1	18
7	REN	17
8	PTPRC	17
9	ITGB1	16
10	BCL2L1	16
11	ACE	15
12	PLG	15
13	MMP3	13
14	IGF1R	13
15	PRKCA	12
16	CTSB	11
17	XIAP	11
18	ITGB3	11
19	F2	10
20	NR3C1	10
21	CASP1	10

**Table 3:** KEGG pathway enrichment analysis.

ID	Signaling Pathway	Enriched Gene Number	*p-*value
hsa04933	AGE-RAGE signaling pathway	5	0.000238979
hsa04668	TNF signaling pathway	5	0.000404789
hsa04151	PI3K-akt signaling pathway	8	0.000751101
hsa04926	Relaxin signaling pathway	5	0.000772967
hsa04015	Rap1 signaling pathway	6	0.001119119
hsa04012	ErbB signaling pathway	4	0.001321844
hsa04912	GnRH signaling pathway	4	0.001844088
hsa04657	IL-17 signaling pathway	4	0.001918105
hsa04625	C-type lectin receptor signaling pathway	4	0.002775155
hsa04066	HIF-1 signaling pathway	4	0.003289067
hsa04621	NOD-like receptor signaling pathway	5	0.003705682
hsa04370	VEGF signaling pathway	3	0.004472814
hsa04919	Thyroid hormone signaling pathway	4	0.0047801
hsa04068	FoxO signaling pathway	4	0.006326533
hsa04917	Prolactin signaling pathway	3	0.007211213
hsa04024	cAMP signaling pathway	5	0.007706803
hsa04921	Oxytocin signaling pathway	4	0.011068984
hsa05022	Pathways of neurodegeneration	7	0.016957114

**Table 4:** Molecular interactions of key targets with AS6.

Compound	Target	PDB ID	Affinity (kcal/mol)	Number of hydrogen bonds	Hydrogen bonds interacting residues
Asperosaponin VI	JUN	5FV8	−7.2	6	Gln-30 (2), Arg-5, Arg-21, Asp-26, Lys-22
Asperosaponin VI	CASP3	3DEI	−9.7	6	Arg-164 (4), Cys-264, Glu-124
Asperosaponin VI	STAT3	6NUQ	−7.6	5	Glu-324 (2), Ser-513, Gln-247, Cys-251
Asperosaponin VI	SRC	2SRC	−9.3	9	Glu-339 (2), His-319, Gln-253, Lys-152, Phe-150, Tyr-90, Thr-247, Ser-248
Asperosaponin VI	PTGS2	5F19	−10.5	3	Glu-236, Ser-143, Glu-140

## Data Availability

The data to support the study's results came from the first author.

## Supplementary Materials

Supplementary Materials Supplementary Table S1. The Structure of Asperosaponin VI. Supplementary Table S2. Targets of Asperosaponin VI.

## References

[1] Practice Committee of the American Society for Reproductive Medicine (2013). Definitions of infertility and recurrent pregnancy loss: a committee opinion. *Fertility and Sterility*.

[2] Kuon R. J., Strowitzki T., Sohn C., Daniel V., Toth B. (2015). Immune profiling in patients with recurrent miscarriage. *Journal of Reproductive Immunology*.

[3] El Hachem H., Crepaux V., May-Panloup P., Descamps P., Legendre G., Bouet P. E. (2017). Recurrent pregnancy loss: current perspectives. *International Journal of Women’s Health*.

[4] Quintero-Ronderos P., Laissue P. (2020). Genetic Variants contributing to early recurrent pregnancy loss etiology identified by sequencing approaches. *Reproductive Sciences*.

[5] Gu H., Li L., Du M. (2021). Key gene and functional pathways identified in unexplained recurrent spontaneous abortion using targeted RNA sequencing and clinical analysis. *Frontiers in Immunology*.

[6] van den Berg M. M. J., van Maarle M. C., van Wely M., Goddijn M. (2012). Genetics of early miscarriage. *Biochimica et Biophysica Acta*.

[7] Shah M. S., Cinnioglu C., Maisenbacher M., Comstock I., Kort J., Lathi R. B. (2017). Comparison of cytogenetics and molecular karyotyping for chromosome testing of miscarriage specimens. *Fertility and Sterility*.

[8] McQueen D. B., Lathi R. B. (2019). Miscarriage chromosome testing: indications, benefits and methodologies. *Seminars in Perinatology*.

[9] Awolumate O. J., Kang A., Khokale R., Cancarevic I. (2020). Role of low molecular weight heparin in the management of unexplained recurrent pregnancy loss: a review of literature. *Cureus*.

[10] Li H. F., Shen Q. H., Li X. Q. (2020). The efficacy of traditional Chinese medicine Shoutai pill combined with western medicine in the first trimester of pregnancy in women with unexplained recurrent spontaneous abortion: a systematic review and meta-analysis. *BioMed Research International*.

[11] Cao L., Chen H., Huang Y., Chen L., Kang M., Liang J. (2020). The pharmacological activity of the wenjing decoction in recurrent spontaneous abortion. *Evidence-based Complementary and Alternative Medicine*.

[12] Li Y. Q., Li W. L., Yu X. H. (2021). Mechanisms of Traditional Chinese Medicine Bushenantai granules in promoting angiogenesis at the maternal-fetal interface of recurrent spontaneous abortion mice. *Journal of Traditional Chinese Medicine*.

[13] Zhang J., Chen L., Zheng C. H., Wang J., Xie D., Zhou Y. X. (2019). Effect of Shoutai pills on Th1/Th2 cytokines in serum and endometrium of rats with stimulated ovulation. *Current Medical Science*.

[14] Tao Y., Chen L., Yan J. (2020). Traditional uses, processing methods, phytochemistry, pharmacology and quality control of Dipsacus asper Wall. ex C. B. Clarke: a review. *Journal of Ethnopharmacology*.

[15] Yang M., Luo J., Yang Q., Xu L. (2021). Research on the medication rules of Chinese herbal formulas on treatment of threatened abortion. *Complementary Therapies in Clinical Practice*.

[16] Xiao T. T., Xu M., Yang X. H. (2014). The evaluation on embryotoxicity of Dipsaci Radix with mice and embryonic stem cells. *Journal of Ethnopharmacology*.

[17] Gao J., Zhou C., Li Y. (2016). Asperosaponin VI promotes progesterone receptor expression in decidual cells via the notch signaling pathway. *Fitoterapia*.

[18] Wang C. G., Lou Y. T., Tong M. J. (2018). Asperosaponin VI promotes angiogenesis and accelerates wound healing in rats via up-regulating HIF-1*α*/VEGF signaling. *Acta Pharmacologica Sinica*.

[19] Zhang J., Yi S., Xiao C. (2020). Asperosaponin VI inhibits LPS-induced inflammatory response by activating PPAR-gamma pathway in primary microglia. *Saudi Journal of Biological Sciences*.

[20] Tang F., Zhang P., Zhao W. (2022). Research on the mechanism of kaempferol for treating senile osteoporosis by network pharmacology and molecular docking. *Evidence-Based Complementary and Alternative Medicine*.

[21] Ru J., Li P., Wang J. (2014). TCMSP: a database of systems pharmacology for drug discovery from herbal medicines. *Journal of Cheminformatics*.

[22] Gfeller D., Grosdidier A., Wirth M., Daina A., Michielin O., Zoete V. (2014). SwissTargetPrediction: a web server for target prediction of bioactive small molecules. *Nucleic Acids Research*.

[23] Stelzer G., Rosen N., Plaschkes I. (2016). The GeneCards suite: from gene data mining to disease genome sequence analyses. *Current Protocols in Bioinformatics*.

[24] Amberger J. S., Hamosh A. (2017). Searching online mendelian inheritance in man (OMIM): a knowledgebase of human genes and genetic phenotypes. *Current Protocols in Bioinformatics*.

[25] Mering C. v., Huynen M., Jaeggi D., Schmidt S., Bork P., Snel B. (2003). STRING: a database of predicted functional associations between proteins. *Nucleic Acids Research*.

[26] Shannon P., Markiel A., Ozier O. (2003). Cytoscape: a software environment for integrated models of biomolecular interaction networks. *Genome Research*.

[27] Trott O., Olson A. J. (2010). AutoDock Vina: improving the speed and accuracy of docking with a new scoring function, efficient optimization, and multithreading. *Journal of Computational Chemistry*.

[28] Ku C. W., Zhang X., Zhang V. R. Y. (2021). Gestational age-specific normative values and determinants of serum progesterone through the first trimester of pregnancy. *Scientific Reports*.

[29] Schmidt-Hansen M., Hawkins J. E., Lord J. (2020). Long-acting reversible contraception immediately after medical abortion: systematic review with meta-analyses. *Human Reproduction Update*.

[30] Zheng Q., Yang Y., Cui X., Zhang D., Liu S., Yan Q. (2018). AP1 mediates uPA/uPAR induced FUT4 expression and trophoblast invasion. *Journal of Cellular Biochemistry*.

[31] Zhang Y., Wang Y., Wang X. H., Zhou W. J., Jin L. P., Li M. Q. (2018). Crosstalk between human endometrial stromal cells and decidual NK cells promotes decidualization in vitro by upregulating IL25. *Molecular Medicine Reports*.

[32] Liu X., Zhao J., Luan X., Li S., Zhai J., Du Y. (2020). SPARCL1 impedes trophoblast migration and invasion by down-regulating ERK phosphorylation and AP-1 production and altering EMT-related molecule expression. *Placenta*.

[33] Li M., Wu Z. M., Yang H., Huang S. J. (2011). NF*κ*B and JNK/MAPK activation mediates the production of major macrophage- or dendritic cell-recruiting chemokine in human first trimester decidual cells in response to proinflammatory stimuli. *The Journal of Clinical Endocrinology and Metabolism*.

[34] Huang S. J., Chen C. P., Buchwalder L. (2019). Regulation of CX3CL1 expression in human first-trimester decidual cells: implications for preeclampsia. *Reproductive Sciences*.

[35] El-Sheikh Ali H., Loux S. C., Kennedy L. (2021). Transcriptomic analysis of equine chorioallantois reveals immune networks and molecular mechanisms involved in nocardioform placentitis. *Veterinary Research*.

[36] Kyathanahalli C., Organ K., Moreci R. S. (2015). Uterine endoplasmic reticulum stress-unfolded protein response regulation of gestational length is caspase-3 and -7-dependent. *Proceedings of the National Academy of Sciences of the United States of America*.

[37] Li C., Zhang X., Kang X. (2020). Upregulated TRAIL and reduced DcR2 mediate apoptosis of decidual PMN-MDSC in unexplained recurrent pregnancy loss. *Frontiers in Immunology*.

[38] Yu H. F., Zheng L. W., Yang Z. Q. (2021). TAZ as a novel regulator of oxidative damage in decidualization via Nrf2/ARE/Foxo1 pathway. *Experimental and Molecular Medicine*.

[39] Verma P., Verma R., Nair R. R. (2019). Altered crosstalk of estradiol and progesterone with Myeloid-derived suppressor cells and Th1/Th2 cytokines in early miscarriage is associated with early breakdown of maternal-fetal tolerance. *American Journal of Reproductive Immunology*.

[40] Teng C. B., Diao H. L., Ma X. H., Xu L. B., Yang Z. M. (2004). Differential expression and activation of Stat3 during mouse embryo implantation and decidualization. *Molecular Reproduction and Development*.

[41] Zong S., Li C., Luo C. (2016). Dysregulated expression of Ido may cause unexplained recurrent spontaneous abortion through suppression of trophoblast cell proliferation and migration. *Scientific Reports*.

[42] Zhu X. X., Yin X. Q., Hei G. Z. (2021). Increased miR-6875-5p inhibits plasmacytoid dendritic cell differentiation via the STAT3/E2-2 pathway in recurrent spontaneous abortion. *Molecular Human Reproduction*.

[43] Deng W., Yuan J., Cha J. (2019). Endothelial cells in the decidual bed are potential therapeutic targets for preterm birth prevention. *Cell Reports*.

[44] Wieser F., Schneeberger C., Hudelist G. (2002). Endometrial nuclear receptor co-factors SRC-1 and N-CoR are increased in human endometrium during menstruation. *Molecular Human Reproduction*.

[45] Jeong J. W., Lee K. Y., Han S. J. (2007). The p160 steroid receptor coactivator 2, SRC-2, regulates murine endometrial function and regulates progesterone-independent and -dependent gene expression. *Endocrinology*.

[46] Szwarc M. M., Hai L., Gibbons W. E. (2018). Retinoid signaling controlled by SRC-2 in decidualization revealed by transcriptomics. *Reproduction*.

[47] Wu H. M., Huang H. Y., Soong Y. K., Leung P. C. K., Wang H. S. (2019). Kisspeptin regulation of human decidual stromal cells motility via FAK-Src intracellular tyrosine kinases. *Human Reproduction*.

[48] Zhu Y.-Y., Wu Y., Chen S.-T. (2021). In situ synthesized monosodium urate crystal enhances endometrium decidualization via sterile inflammation during pregnancy. *Frontiers in Cell and Developmental Biology*.

[49] Sales K. J., Grant V., Catalano R. D., Jabbour H. N. (2011). Chorionic gonadotrophin regulates CXCR4 expression in human endometrium via E-series prostanoid receptor 2 signalling to PI3K-ERK1/2: implications for fetal-maternal crosstalk for embryo implantation. *Molecular Human Reproduction*.

[50] Zhang D., Chang X., Bai J., Chen Z. J., Li W. P., Zhang C. (2016). The study of cyclooxygenase 2 in human decidua of preeclampsia. *Biology of Reproduction*.

[51] Zhao H., Hu S., Qi J. (2022). Increased expression of HOXA11-AS attenuates endometrial decidualization in recurrent implantation failure patients. *Molecular Therapy*.

[52] Kohlmeier A., Sison C. A. M., Yilmaz B. D., Coon V J. S., Dyson M. T., Bulun S. E. (2020). GATA2 and progesterone receptor interaction in endometrial stromal cells undergoing decidualization. *Endocrinology*.

[53] Zhou M., Xu H., Zhang D. (2021). Decreased PIBF1/IL6/p-STAT3 during the mid-secretory phase inhibits human endometrial stromal cell proliferation and decidualization. *Journal of Advanced Research*.

[54] Zhou W. J., Yang H. L., Mei J. (2022). Fructose-1,6-bisphosphate prevents pregnancy loss by inducing decidual COX-2(+) macrophage differentiation. *Science Advances*.

[55] Yang D., Ding J., Wang Y. (2020). YY1-PVT1 affects trophoblast invasion and adhesion by regulating mTOR pathway-mediated autophagy. *Journal of Cellular Physiology*.

[56] Vural P., Akgul C., Yildirim A., Canbaz M. (2000). Antioxidant defence in recurrent abortion. *Clinica Chimica Acta*.

[57] Li Z., Zhou G., Jiang L., Xiang H., Cao Y. (2019). Effect of STOX1 on recurrent spontaneous abortion by regulating trophoblast cell proliferation and migration via the PI3K/AKT signaling pathway. *Journal of Cellular Biochemistry*.

[58] Mei J., Yan Y., Li S. Y. (2019). CXCL16/CXCR6 interaction promotes endometrial decidualization via the PI3K/AKT pathway. *Reproduction*.

[59] Arck P. C., Troutt A. B., Clark D. A. (1997). Soluble receptors neutralizing TNF-alpha and IL-1 block stress-triggered murine abortion. *American Journal of Reproductive Immunology*.

[60] Wang W. J., Liu F. J., Xin L. (2014). Adoptive transfer of pregnancy-induced CD4+CD25+ regulatory T cells reverses the increase in abortion rate caused by interleukin 17 in the CBA/JxBALB/c mouse model. *Human Reproduction*.

