# Supplementary material for: Research on the Mechanism of Asperosaponin VI for Treating Recurrent Spontaneous Abortion by Bioinformatics Analysis and Experimental Validation
Source: Evid Based Complement Alternat Med. 2022 Jun 23;2022:8099853. doi: 10.1155/2022/8099853 (PMC9246589; doi:10.1155/2022/8099853)
Supplement: Supplementary Materials — Supplementary Table S1. The Structure of Asperosaponin VI. Supplementary Table S2. Targets of Asperosaponin VI. [file 8099853.f1.docx]

| Molecule ID | PubChem CID | Molecule name | Chemical formula | Structure |
| --- | --- | --- | --- | --- |
| MOL003106 | 118705380 | Asperosaponin VI | C_47_H_76_O_18_ | 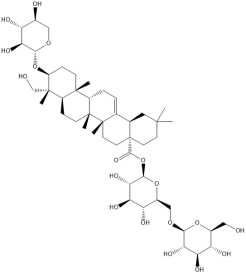 |

**Supplementary Table S1. The Structure of Asperosaponin VI**

**Supplementary Table S2. Targets of Asperosaponin VI**

| Number | Gene symbol | Number | Gene symbol | Number | Gene symbol |
| --- | --- | --- | --- | --- | --- |
| 1 | BCL2L1 | 36 | HTR2B | 71 | CASP1 |
| 2 | F2 | 37 | ADRA2A | 72 | PTPN2 |
| 3 | RORC | 38 | ADRA2C | 73 | CAPN1 |
| 4 | PTPA | 39 | ADRA2B | 74 | PDE5A |
| 5 | GLI1 | 40 | DRD1 | 75 | GRB2 |
| 6 | STAT3 | 41 | DRD2 | 76 | PDE6A |
| 7 | PPP1CC | 42 | DRD3 | 77 | PRKCA |
| 8 | GLRA1 | 43 | CYP2D6 | 78 | ACE |
| 9 | GLRA2 | 44 | HTR6 | 79 | LTB4R |
| 10 | TYMS | 45 | HTR1B | 80 | CMA1 |
| 11 | ADORA3 | 46 | RRM1 | 81 | ITGAV |
| 12 | PTPN1 | 47 | ADK | 82 | HLA-A |
| 13 | PRKCD | 48 | ATP1A1 | 83 | BIRC3 |
| 14 | PRKCQ | 49 | TBXAS1 | 84 | PTPRS |
| 15 | SLC5A2 | 50 | PPM1A | 85 | MMP13 |
| 16 | SLC5A1 | 51 | LCK | 86 | MMP3 |
| 17 | ADORA1 | 52 | ADRB1 | 87 | MMP10 |
| 18 | SLC28A2 | 53 | NR3C2 | 88 | SIRT1 |
| 19 | PPM1B | 54 | NR3C1 | 89 | MMP8 |
| 20 | PPP2R5A | 55 | SRC | 90 | APEX1 |
| 21 | TOP1 | 56 | XIAP | 91 | CHIA |
| 22 | ADORA2A | 57 | PLG | 92 | HDAC6 |
| 23 | PTGS2 | 58 | ITGB1 | 93 | IGF1R |
| 24 | F2RL1 | 59 | NOS2 | 94 | HDAC8 |
| 25 | PTAFR | 60 | LIPC | 95 | HDAC1 |
| 26 | HSD11B2 | 61 | PTPRC | 96 | TMPRSS11D |
| 27 | HSD11B1 | 62 | LIPG | 97 | EIF4A1 |
| 28 | PRKCG | 63 | F3 | 98 | SLC10A2 |
| 29 | PRKCH | 64 | LNPEP | 99 | ST14 |
| 30 | GBA | 65 | PTPN22 | 100 | ADAM17 |
| 31 | PTPRA | 66 | CTSB | 101 | ITGA4 |
| 32 | JUN | 67 | CASP4 | 102 | ITGB3 |
| 33 | VAV1 | 68 | CASP3 | 103 | F7 |
| 34 | TRPV4 | 69 | REN |  |  |
| 35 | VDR | 70 | CASP7 |  |  |
